# Ultra-intense pulsed source of ionizing radiation based on direct laser acceleration of electrons for studying the FLASH effect

**DOI:** 10.1038/s41598-026-40281-4

**Published:** 2026-02-17

**Authors:** Mikhail Gyrdymov, Nikolai Bukharskii, Vratislav Fabian, Michael Häfner, Pharewa Karoon, Nataliya G. Borisenko, Jakub Cikhardt, Sero Zähter, Philipp Korneev, Joachim Jacoby, Nikolay E. Andreev, Olga N. Rosmej

**Affiliations:** 1 Institute for Applied Physics (IAP), Goethe University Frankfurt, Frankfurt am Main, Germany; 2 GSI Helmholtzzentrum für Schwerionenforschung, Darmstadt, Germany; 3 P. N. Lebedev Physical Institute (LPI), Russian Academy of Sciences, Moscow, Russia; 4 National Research Nuclear University MEPhI, Moscow, Russia; 5 Faculty of Electrical Engineering, Czech Technical University in Prague, Prague, Czechia; 6 School of Physics, Institute of Science, Suranaree University of Technology, Nakhon Ratchasima, Thailand; 7 Focused Energy GmbH, Darmstadt, Germany; 8 Helmholtz Forschungsakademie Hessen für FAIR, Frankfurt am Main, Germany; 9 Joint Institute for High Temperatures, Russian Academy of Sciences, Moscow, Russia; 10 Moscow Institute of Physics and Technology (State University), Dolgoprudny, Russia

**Keywords:** Optics and photonics, Physics

## Abstract

Results of the pilot experiment on the ultra-intense irradiation of a water phantom and various biological media with laser-driven beams of relativistic electrons in context of the FLASH effect are presented. Directed high-current beams of MeV electrons were generated in the interaction of a sub-picosecond PHELIX laser pulse at 10^19^ W/cm^2^ intensity with a low-density polymer foam, which was converted into a plasma of near-critical density by a preceding nanosecond laser pulse. The combination of 20–50 Gy of ionizing radiation delivered by the relativistic electron beam in a single laser shot and the world’s highest dose rate of 70 Gy/ps makes this source unique for studying the FLASH effect and for applications. The picosecond duration of the electron beam allows for separation of the process of ultrafast (instantaneous) oxygen ionization and the subsequent chemical reactions. In each laser shot, a sudden drop in the molecular oxygen concentration as a function of the delivered dose was measured in water and biological media. The dependence obtained for water is consistent with the results of the Monte Carlo simulations.

**Supplementary Information:**

The online version contains supplementary material available at 10.1038/s41598-026-40281-4.

## Introduction

Modern radiotherapy employs high-energy photons, protons, and electrons to treat cancer, but collateral damage to healthy tissue remains a major clinical limitation. Low linear energy transfer (LET) radiation such as X-rays and electrons induces DNA damage predominantly through the radiolysis of water, leading to the formation of reactive oxygen species (ROS). The presence of oxygen in normal tissues amplifies this effect — a phenomenon known as the oxygen effect — which contributes to radiation-induced toxicities such as inflammation and fibrosis^1,2^. To improve the therapeutic ratio, innovative approaches are being pursued to reduce normal tissue injury without compromising tumor control.

One promising strategy is the ultra-high dose-rate (UHDR) irradiation, known as FLASH radiotherapy, which delivers a therapeutic dose within a fraction of a second (typically > 40 Gy/s). Preclinical studies have shown that FLASH can significantly spare normal tissue while maintaining tumor control — a phenomenon referred to as the FLASH effect^3–9^. Although the underlying mechanisms are not fully understood, it is hypothesized that rapid radiolytic depletion of oxygen and altered ROS dynamics contribute to this transient radioresistance.

A novel X-ray UHDR system is proposed^10^based on the MeV bremsstrahlung generated in the process of interaction of 12 MeV linac electron beam with a converter. According to Monte Carlo simulations, the expected dose rate can reach 300 Gy/s, with the accumulated absolute dose of 10 Gy, which is sufficient for therapeutic FLASH effect. Achieving FLASH-compatible dose rates remains a technical challenge due to the limitation of the dose rate of ionizing radiation in conventional accelerators with pulse durations in the nanosecond to sub-second range. It has sparked interest in alternative irradiation systems, such as high-power lasers that can generate X-ray and particle beams with dose rates exceeding 10^9^ Gy/s.

The irradiation with protons performed in the DRACO laser single-shot regime, delivered single bunches with the dose in the range of 6–28 Gy and mean dose rates of ∼ 10^9^ Gy/s^11^. The generation of MA current-directed beams of relativistic electrons by direct laser acceleration was demonstrated at the PHELIX laser (**P**etawatt **H**igh **E**nergy **L**aser for Heavy **I**on E**x**periments)^12^that provides a unique platform for investigating FLASH effects under controlled conditions.

Since biological tissues consist primarily of water, radiation-induced chemical processes in vivo are largely mediated by water radiolysis and the subsequent formation of ROS, such as hydroxyl radicals (·OH), superoxide anions (O₂·⁻), and hydrogen peroxide (H₂O₂)^13–16^. These reactive species account for the majority of indirect radiation damage to DNA, which can be described/simulated/predicted using Monte Carlo track-structure codes^14^. Of particular interest is the role of inter-track radical recombination under UHDR, a phenomenon that has been theoretically anticipated but remains experimentally unverified.

Typical benchmarking scenarios involve ultra-short irradiation in aqueous systems or solutions containing homogeneously distributed target molecules. However, experimental data under such conditions are scarce, especially in oxygenated environments, which are representative of biological systems. A major challenge lies in detecting measurable radical concentrations on sub-microsecond timescales – the critical window for radiation-induced chemical transformations, see Table 1.

In Table 1, the stages dominated by the key processes in biological media or aqueous solutions following radiation exposure are shown. Upon irradiation of aqueous systems, ionization and electronic excitation initiate molecular dissociations, resulting in the formation of primary radiolytic species such as radicals and hydrated electrons. These reactive species subsequently diffuse and interact with each other, with the surrounding water, and with dissolved molecular oxygen or biomolecules during the expansion of the chemical track.

**Table 1:** Stages dominated by the key processes in biological media or aqueous solutions following radiation exposure (Adapted from Fig. 2 in ^17^).

Stage	Processes in biological media or aqueous solutions	Characteristic duration [s]
Physical	Primary ionization, excitation, transport of secondary electrons	10^−17^ – 10^−15^
Pre-chemical	Dissociation of excited / ionized molecules	10^−15^ – 10^−12^
Chemical (heterogeneous)	Diffusion and reaction of generated radical species	10^−12^ – 10^−6^
Homogeneous chemistry	Further chemical reactions (no memory of initial chemical track)	10^−6^ – 1
Biochemical	Enzymatic repair processes	1–10^3^
Biological	Cellular and tissue response	10^3^ – 10^5^

The use of laser-accelerated electron bunches with durations on the order of 1 picosecond enables separation between the ultrafast ionization of oxygen – occurring at the end of the physical stage and onset of the pre-chemical stage – and subsequent chemical reactions. This temporal decoupling presents a significant advantage for Monte Carlo simulations, such as those performed with the TRAX-CHEM code, which can model these conditions with high fidelity^14^.

The amount of oxygen consumed during irradiation is directly linked to the yield of radiation-induced radical species and can therefore serve as a benchmark for Monte Carlo-based simulation tools.

In our pilot experiment, we demonstrate – for the first time – the applicability of this novel ionizing radiation source for radiochemical investigations. Specifically, we quantified the rate of oxygen consumption via the key reaction channels e_aq_⁻ + O_2_ → O_2_⁻ and H**·** + O_2_ → HO_2_ in both pure water and biologically relevant aqueous media at instantaneous dose rates of 50–70 Gy/ps and per shot doses of up to 50 Gy – a uniquely high-value achievement in this context.

The results of this study – assuming further improvements in dosimetric accuracy – pave the way for benchmarking the chemical-stage predictions of Monte Carlo track-structure codes and radio-therapeutic applications of laser-driven relativistic electrons.

### Direct laser acceleration of electrons

Laser-driven sources of particles and X-rays enable instantaneous dose rates that far exceed those achievable with standard technologies^18^, thus offering new opportunities for exploring the biological and chemical underpinnings of the FLASH effect^19^. Among laser-driven ionization sources, relativistic electron beams offer advantageous characteristics: higher energy deposition per unit path length compared to X-rays, while achieving more uniform volumetric energy deposition compared to protons and ions.

In this study, we demonstrate that the acceleration of electrons in low-density polymer foam, enabled by the 200 TW short-pulse PHELIX laser system^12,20,21^, provides a unique platform for generating highly charged, collimated MeV-range electron beams capable of depositing several tens of Gy in biological targets within a single picosecond-scale laser shot.

Direct laser acceleration (DLA) of electrons in plasmas of near-critical electron density (NCD) offers a promising mechanism for generating ultra-high dose rate ionizing radiation. During this process, the relativistic laser pulse propagates through the plasma, forming a plasma channel characterized by a significant radial inhomogeneity of the electron density. The ponderomotive expulsion of electrons from the channel axis creates a quasi-static radial electrostatic field, while the current of accelerated particles simultaneously generates a strong azimuthal magnetic field. Electrons trapped within this channel undergo transverse betatron oscillations; they efficiently gain energy from the laser field when the frequency of these oscillations resonates with the Doppler shifted laser frequency^22,23^. Depending on the specific interplay between laser intensity and plasma density, the acceleration can be driven by DLA, self-modulated laser wakefield acceleration (SM-LWFA), stochastic heating^24^, or a synergy of these processes.

Measurements and numerical simulations confirmed that the DLA-generated electron beam is well-collimated and its energy distribution follows an exponential function characterized by an effective temperature up to 10–15 times exceeding those achieved with conventional solid-foil targets irradiated under comparable laser conditions^12,20,21,25^.

The reported experiment was conducted at the PHELIX laser facility (GSI Helmholtzzentrum Darmstadt)^26^in a single-pulse mode with the highest nanosecond laser contrast of ≥ 10^11^. A s-polarized laser pulse of 1.053 μm fundamental wavelength and 750 ± 250 fs duration delivered by the Nd: glass laser was sent onto the target at 3° to the target normal. The laser energy of 70 ± 10 J measured before the main amplifier was focused by means of a 150 cm off-axis parabolic mirror into a slightly elliptical focal spot of 12 ± 2 μm and 15 ± 2 μm containing *E*_*FWHM*_ ≃ 17 ± 3 J at FWHM. Resulting peak intensity reached (1–2) ×10^19^ W/cm^2^.

Polymer CHO aerogels with an initial average density of 2–5 mg/cm³ and a thickness of 300–1500 μm, grown inside a copper washer with the inner diameter of 2.5 mm, served as targets^27^. Upon complete ionization, the electron density of the resulting plasma corresponded to 0.65 n_cr_, with a critical electron density of n_cr_ = 10^21^ cm^− 3^ for λ = 1.053 μm. This means that relativistic laser pulse can propagate in such plasma without reflection.

A rectangular pulse of 3 ns duration, preceded a short pulse of relativistic intensity, was used to convert the foam layer into plasma. The nanosecond and sub-ps pulses were focused onto the target by the same off-axis parabolic mirror. Depending on the foam density and thickness, the intensity of the pre-ionized ns-pulse was (1–7) ×10¹³ W/cm² and the delay between the ns and ps pulses were adjusted to achieve complete foam ionization and homogenization^25^. After a delay of 3–5 ns, the sub-ps laser pulse interacted with the plasma target.

When applying a nanosecond pulse with an intensity of ~ 10¹³ W/cm², the plasma profile at the moment of arrival of the sub-ps pulse contains an under-dense region and a region with a near-critical electron density (NCD)^25,28^. The most intense energy gain by electrons due to direct laser acceleration occurs in the region of a sharp increase in density and in the NCD region, as shown in^25^. The stability of this scheme was demonstrated experimentally^12,29^and in simulations using different plasma profiles as input data in 3D PIC simulations^25^.

**Fig. 1:**
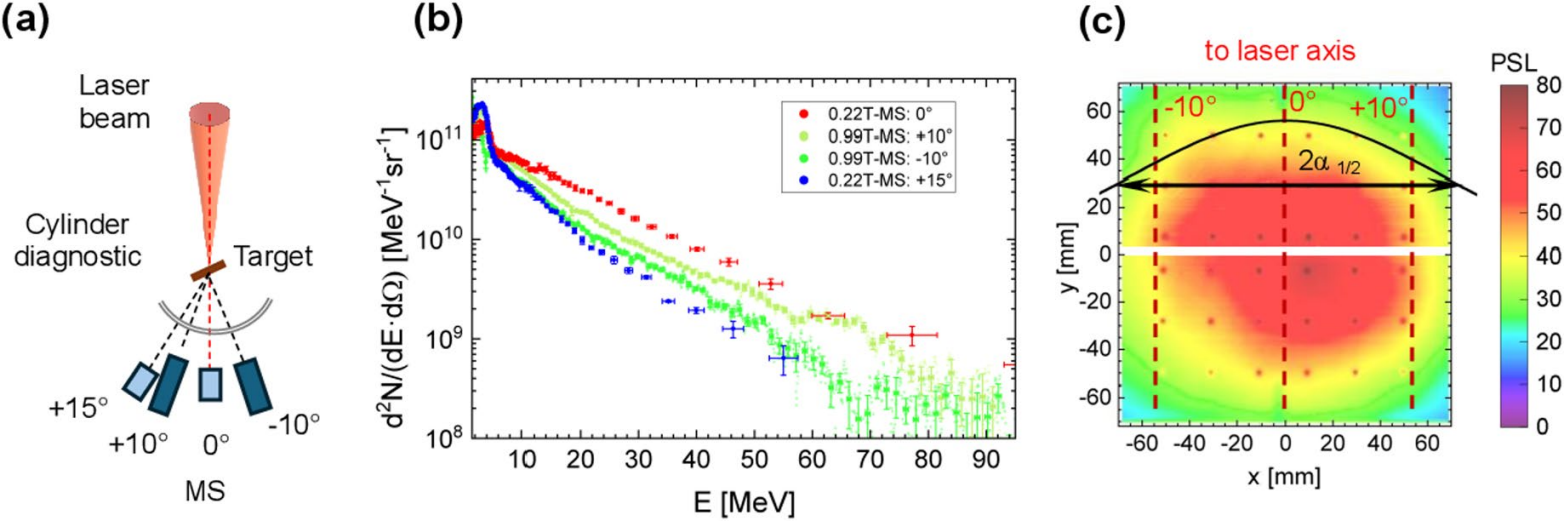
(**a**) Experimental setup for measurements of the angular-dependent electron spectra. (**b**) Electron spectra measured for a laser shot on an 800 ± 50 μm thick polymer foam target with a mean density of 2 mg/cm³ (shot #25 P207). (**c**) Angular distribution of electrons with energy E_e_ > 7.5 MeV recorded using a cylindrical diagnostic consisting of a steel stack with imaging plates (IP) inserted between the sheets.

Figure 1a illustrates the experimental setup, used to characterize the properties of the DLA electron beam in the FLASH campaign, which includes up to four 0.99 T magnetic spectrometers charged with imaging plates (IPs) and positioned at various angles relative to the laser axis. Figure 1b shows the measured electron spectra in case of 800 ± 50 μm thick polymer foam targets with a mean density of 2 mg/cm^3^^27^, and Fig. 1c demonstrates the angular distribution of electrons with energies exceeding 7.5 MeV that was recorded using a cylindrical detector composed of three 3 mm steel layers interleaved with IPs^12^. The electron beam displays an exponential spectral shape with effective temperatures of approximately 13 MeV at − 10° and up to 16 MeV along the laser axis, confined within a solid angle of 0.16 sr in the case of electrons with energies > 7.5 MeV (5-times ponderomotive potential at 10^19^ W/cm^2^ laser intensity). This results in 33 ± 5 nC charge of directed beam. The picosecond duration of the electron pulse closely matches the laser pulse length^29^, enabling temporally confined ultra-high dose delivery caused by a DLA beam flux of 10^24^ electrons/sr/s.

The experiments conducted at the PHELIX facility demonstrate that such DLA electron beams are capable of delivering radiation doses of 20–50 Gy to aqueous or cellular targets with a single laser pulse at dose rates exceeding 70 Gy/ps.

### Experimental setup for UHDR

For UHDR experiments, airtight cylindrical tanks (water tanks) suitable for operation in vacuum were used. The tanks were filled with various aqueous liquids: deionized water, phosphate-buffered saline (PBS), cell culture medium (F-12 Nutrient Medium, or Ham’s F12), and lysed cells. All samples were fully oxygenated prior to irradiation.

**Fig. 2:**
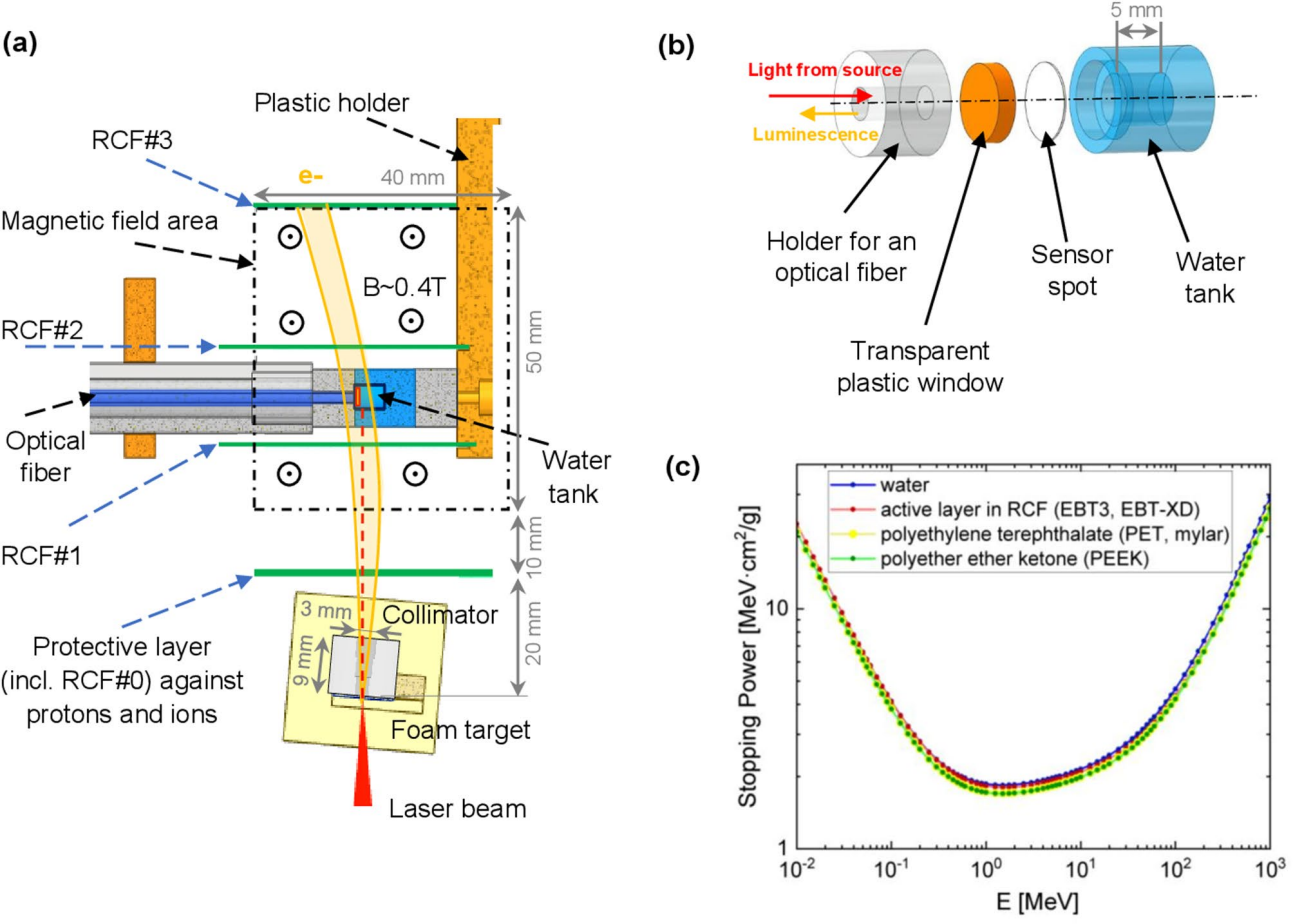
(**a**) Top view of the UHDR setup cross-section at the laser beam level. (**b**) Side view of the water tank setup. (**c**) Electron stopping power in different media used (data from ESTAR^33^).

Figure 2a shows the experimental setup of the water tank irradiation. The schematic representation provides a top view of the UHDR setup cross-section at the laser beam level. The laser beam interacted with a foam target, behind which a carbon collimator with a 20° opening angle was attached. The target plane was rotated at an angle of + 3° clockwise to the laser propagation direction (see Fig. 2a). The collimator was designed to stop or decelerate ponderomotive electrons with energies below 7 MeV emitted at large angles relative to the laser axis. In addition, decelerated electrons were deflected by a 0.4 T magnet placed at 30 mm from the foam target. These measures were intended to reduce the dose distribution non-uniformity within the water tank.

To ensure a dominant dose contribution from high-energy electrons, a protective layer consisting of 150 μm Al foil and three EBT-XD radiochromic films (RCFs) were placed at a distance of 20 mm from the target. This enabled the stopping of protons with energies up to 10 MeV and the attenuation of soft X-rays^30^caused by the betatron radiation of DLA electrons^29^and bremsstrahlung in the carbon collimator.

For quantitative dose measurements, subsequent films RCF#1, RCF#2 and RCF#3 were placed along the particle path, as shown in Fig. 2a.

For molecular oxygen concentration measurements, an oxygen meter was used. It transmitted light at 505 nm through a 2 mm diameter optical fiber to a sensor spot (SP-Pst3-SA23-D5-OIW-US) containing luminophores^31^. The sensor spot was placed on the inner flat wall of the tank filled with the medium, allowing light penetration (Fig. 2b). Due to photoluminescence quenching, oxygen molecules in the medium reduce luminescence from the sensor spot^32^. The resulting signal was sent back to the oxygen meter for analysis, which was connected to a PC for real-time data recording.

To protect the PC and optical sensor from electromagnetic pulses during laser shots, both were housed inside a Faraday cage located adjacent to the target chamber. A remote connection to the PC was established via an internal network for control from the control room.

The tank parts (Fig. 2b) were glued to ensure airtightness, allowing operation under vacuum conditions. The tank walls were made from polyetheretherketone (PEEK), a radiation- and chemically-resistant material that does not leach chemicals or oxygen when irradiated. Before sealing, the tank was completely filled with the sample, ensuring no air bubbles were trapped.

Importantly, the stopping powers of water (or water-like media), the active and protective layers of the RCFs, and PEEK are very similar, as shown in Fig. 2c. This similarity allows us to assume comparable doses within the tank for different contents, provided the experimental conditions – such as target, laser parameters, and tank position – are kept constant. In this regard, the dose reconstruction calculations (see the “Dose reconstruction inside the water tank” section and “Supplementary Information”) were performed using stopping power values taken from ESTAR^33^for Mylar and assumed to be the same for water, RCFs and PEEK layers.

### Dose measurement

The objective of the experiment was to establish a correlation between the decrease in molecular oxygen concentration in the tank, induced by the ultra-high flux laser-driven relativistic electrons, and the corresponding deposited dose. A selected laser shot #9 will be considered below as an example. In this case, DLA electrons were produced via the interaction of a 10^19^ W/cm^2^ PHELIX sub-ps laser pulse with a 1000 μm CHO-foam target of 2 mg/cm³ density, pre-ionized by a 3 ns pulse with Intensity of ~ 3 × 10^13^ W/cm^2^. As direct dose measurements within the tank were not feasible, the dose was reconstructed using RCF data recorded outside the tank, along the electron beam path.

**Fig. 3:**
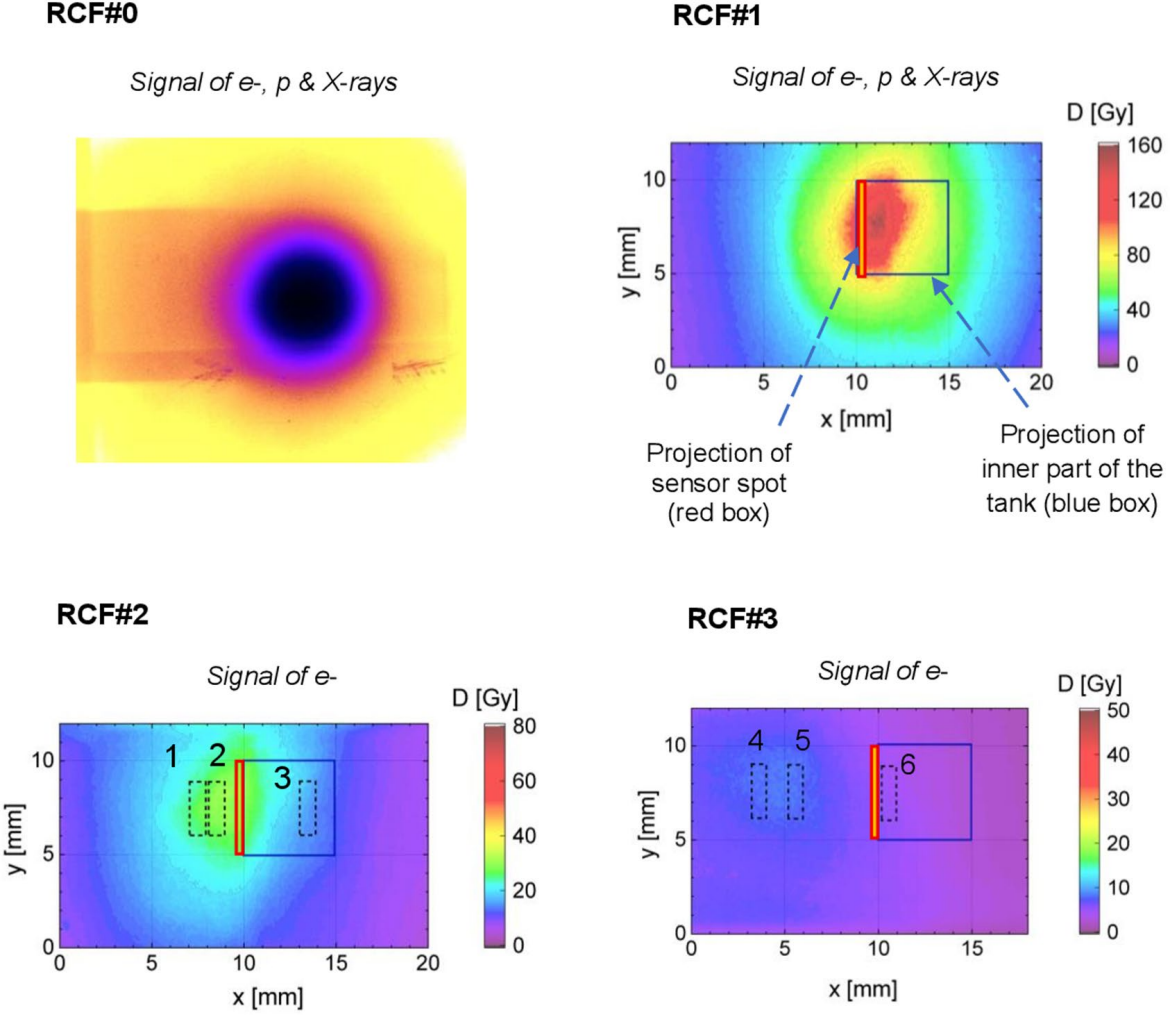
Two-dimensional distributions of the dose recorded on radiochromic films (RCFs): in the protective layer (RCF#0), in front of the tank (RCF#1), behind the tank (RCF#2), and after the magnets (RCF#3).

Figure 3 shows a series of RCFs placed along the paths of the electron beam, X-rays, and protons, illustrating the distribution of the absorbed dose. RCF#0, the second RCF of the protective layer (150 μm Al + 3 RCFs), exhibits a distinct circular imprint caused by bremsstrahlung radiation from electrons passing through the collimator. This radiation provides the dominant contribution to the absorbed dose on RCF#0.

However, the shielding layers were insufficient to stop 16 MeV protons recorded via the Time-of-Flight method^34^, which can reach RCF#1.

Furthermore, although the protective layer significantly attenuates soft X-rays from the primary target, other sources such as betatron radiation (specifically in the 5–10 keV range^29^) and bremsstrahlung within the collimator must also be considered. Consequently, RCF#1 records a combined dose contribution from X-rays, protons, and electrons.

The 2 mm thick PEEK walls of the water tank effectively stop the remaining protons and further attenuate the X-ray flux. Analysis of the dose distribution across the RCF#2 area (e.g., shot #9: peak electron dose ~ 28 Gy, background ~ 1 Gy) indicates that the X-ray contribution is only about 3% of the peak electron dose. For this reason, RCF#2 and RCF#3, located behind the water tank, exhibit a primary contribution from DLA electrons. The lateral displacement of the RCF signal relative to the laser axis (see marked areas 1, 2 on RCF#2 and areas 4, 5 on RCF#3 in Fig. 3) confirms the predominant role of electrons deflected by the 0.4 T static magnetic field.

In Fig. 3, the blue frame indicates the projection of the tank’s inner volume onto the RCFs in the direction perpendicular to the laser beam, while a red slice shows the position of the sensor spot. The dashed regions (1–6) on RCF#2 and RCF#3 are designated for use in the electron spectrum reconstruction method, which is detailed in the section “Dose reconstruction”.

A series of laser shots was performed using foam targets with volume densities of 2–5 mg/cm^3^ and thicknesses ranging from 400 to 1500 μm. Optimized parameters of the ns-pulse used to convert foam into homogeneous plasma were selected for the respective targets (see Table 2).

**Table 2:** Target parameters and optimized laser pulses parameters in the UHDR experiment.

Group	Target		Duration of the ns pulse [ns]	Intensity of pre-ionized ns pulse, ×10^13^ W/cm^2^	Delay sub ps pulse [ns]
	Mean density [mg/cm^3^]	Thickness [µm]			
#1	2	400	3	1	3
#2	2	800–1000	3	3	3
#3	5	500	3	7	5
#4	3	600–700	3	3	3
#5	2	1500	3	3	4

**Fig. 4:**
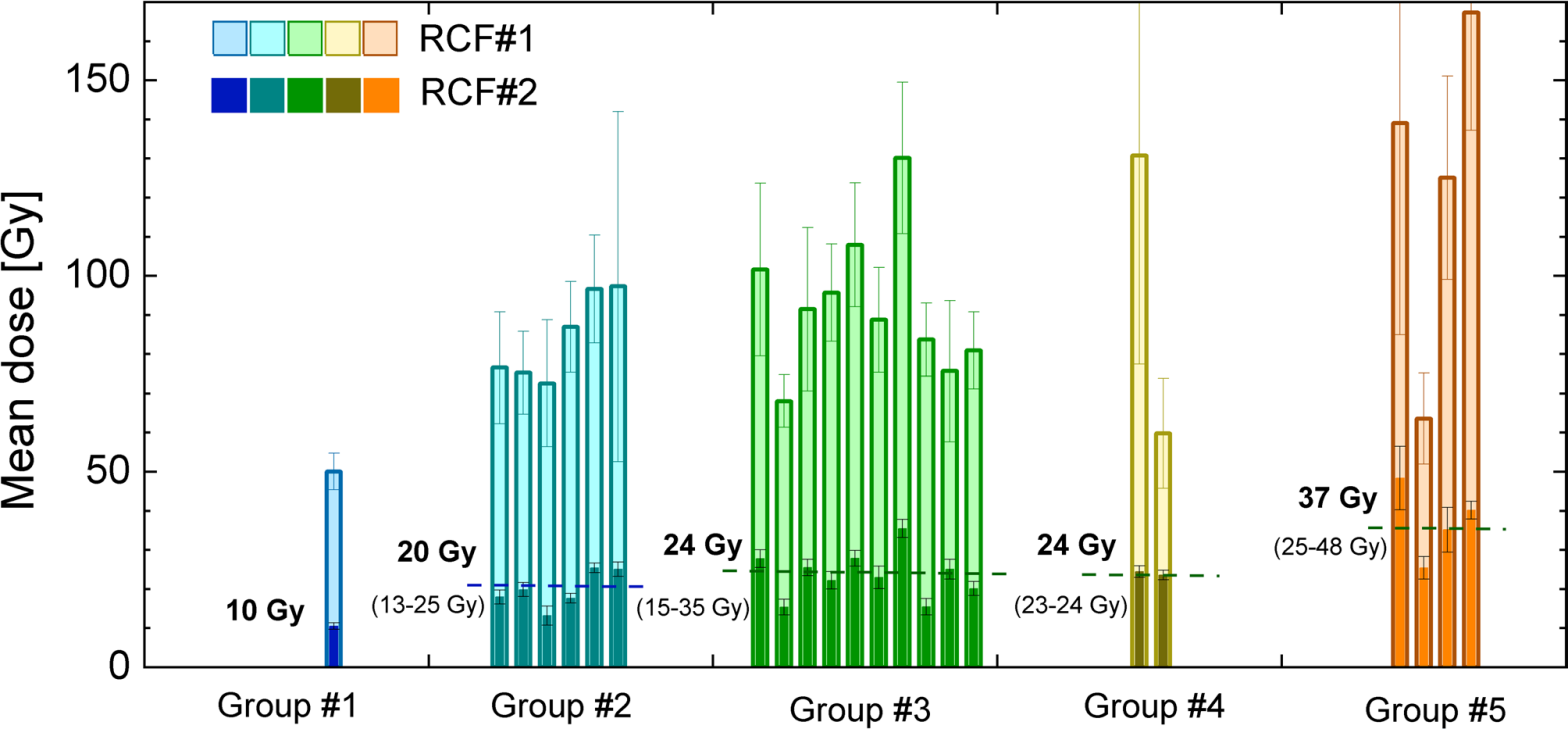
Mean RCF dose within the 5 × 5 mm^2^ interaction area measured in front of the tank (RCF#1) and behind the tank (RCF#2) for various target groups, corresponding to the blue box in Fig. 3.

Figure 4 presents a diagram of doses on RCF#1 caused by protons, X-rays and electrons (indicated by bright bars) and RCF#2 caused by electrons (dark bars), averaged over a 5 × 5 mm² area corresponding to the water tank position (see the “blue box” in Fig. 3). In Fig. 4, laser shots are grouped by similar foam and laser pulse parameters according to the data in Table 2. Comparison of RCF#1 and RCF#2 data indicates that keV X-rays and protons with initial energies exceeding 10 MeV dominate the signal at RCF#1 after passing through the shielding. The dose on RCF#1 increases from approximately 50 Gy to 150 Gy with increasing foam areal density. The relative dose variation due to inhomogeneous distribution in the “blue box” on RCF#1 in front of the tank ranged from 10% to 40% across different laser shots, with the primary contribution from keV X-rays and MeV protons. It can be concluded that the expected dose in water tank takes the value between values for the corresponding area on RCF#1 and RCF#2.

On RCF#2, located behind the tank, the dose originates from DLA electrons, with relative deviations between 5% and 20% due too inhomogeneous dose distribution in the “blue box” across different shots. The RCF#2 dose exhibits the same trend with increasing foam areal density as RCF#1.

The dose differences within each group can be attributed to several factors: laser energy before the compressor varied from 60 to 80 J between shots, and the focusing quality changed significantly over the course of the day due to heating of the Nd: glass gain medium. Furthermore, the uncertainty in the laser intensity is determined by the technical limitations in measuring the laser pulse duration (25%) and the laser energy (10%).

Both the energy and intensity of the laser pulse influence the charge of the DLA electrons and, consequently, the dose of ionizing radiation. However, it has been demonstrated that the DLA process in pre-ionized foam targets is very stable^12,20,25,29^, provided stable laser parameters. Moreover, an extensive series of the experiments reported in^12,20,25,29^has been carried out to optimize the laser parameters for various target conditions discussed in this work.

### Dose reconstruction

In the experiment, simultaneous measurements of the molecular oxygen concentration, the dose inside the tank, and the energy and angular distribution of the DLA electrons were not possible. Therefore, a reconstruction method was developed to estimate the dose at six selected regions (1–6) on RCF#2 and RCF#3 (Fig. 3), based on the dominant dose contribution from DLA electrons. The method involves optimizing the assumed energy and angular distributions of electrons.

From the previous measurements (Fig. 1 and^12,25^), the energy distribution of the DLA electrons can be approximated by an exponential function with two effective temperatures:

1 $$\:f={\frac{{d}^{2}N}{dE\cdot\:d\varOmega\:}|}_{\alpha\:}={N}_{0}\left(\lambda\:\cdot\:exp\left(-\frac{E}{{T}_{1}}\right)\:+(1-\lambda\:)\cdot\:exp\left(-\frac{E}{{T}_{2}}\right)\right)\cdot\:exp\left(-{\left(\frac{\alpha\:-{\alpha\:}_{0}}{\varDelta\:\alpha\:}\right)}^{2}\right)$$,

where $\:{N}_{0}$ is normalization factor in MeV^−1^sr^− 1^, $\:\lambda\:$ and $\:\left(1-\lambda\:\right)$ are the fractions of electrons with effective temperatures $\:{T}_{1}$ and $\:{T}_{2}$, $\:{\alpha\:}_{0}\:$is the angle between the laser axis and the electron beam, and $\:\varDelta\:\alpha\:$ is the divergence parameter related to the half-angle of FWHM: $\:{\varDelta\:\alpha\:}_{1/2FWHM}=\varDelta\:\alpha\:\cdot\:\sqrt{ln\left(2\right)}$ (see Fig. 1c).

As the first step in the dose evaluation, the parameters of Eq. (1) were taken from the reference shot in Fig. 1. Iterative optimization was then performed to determine the six parameters in Eq. (1) that yielded the best match between the calculated and measured doses at all six positions on RCF#2 and RCF#3 shown in Fig. 3 for each laser shot.

The irradiation dose inside the water tank was calculated based on the optimized electron energy distribution, ensuring the best agreement between the measured RCF doses and the corresponding calculated values derived from the stopping power of the RCF. As previously demonstrated in the section “Experimental setup for UHDR” (specifically in Fig. 2c), the electron stopping powers within the water tank and the RCF layers are remarkably similar. This similarity allowed for a significant simplification of the subsequent calculations. Details of the reconstruction method and verification of the results are provided in “Supplementary Information”.

## Results and discussion

**Fig. 5:**
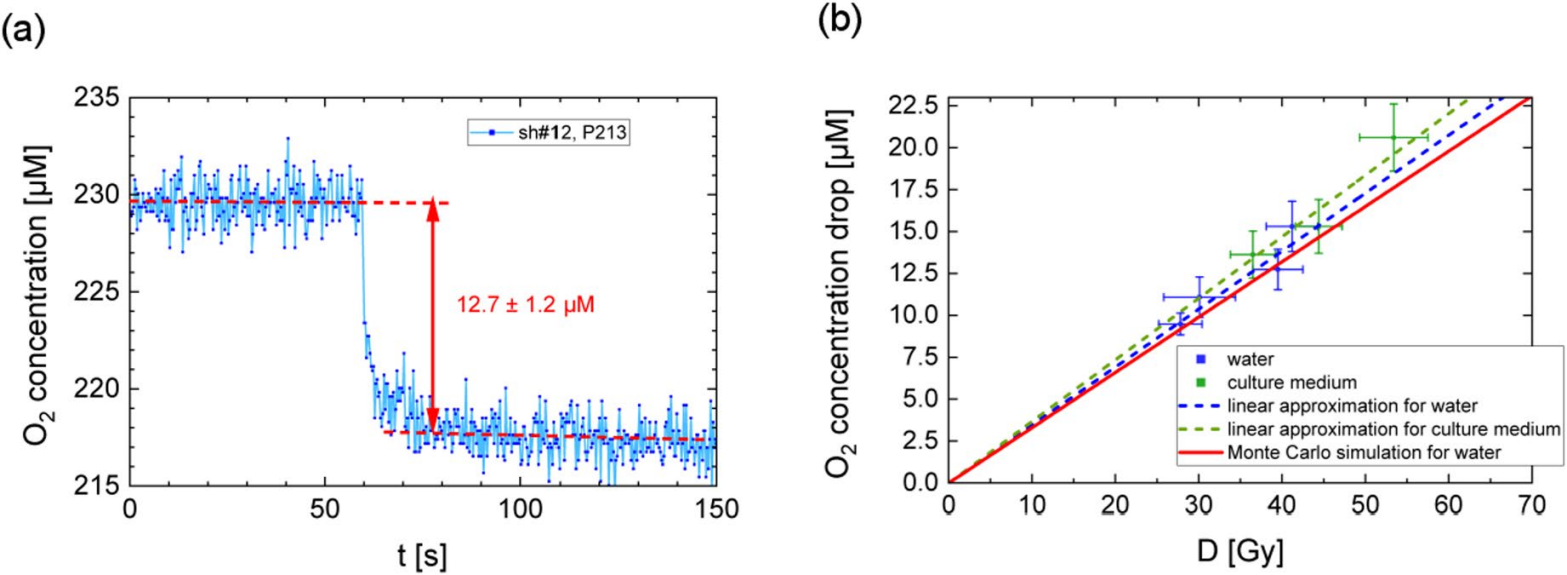
(**a**) Measurement of the molecular oxygen concentration in the tank using an optical fiber probe, shot #12 P213. (**b**) Dependence of the molecular oxygen concentration drop on the dose deposited in media by DLA electrons.

An example of the recorded molecular oxygen concentration drop is shown in Fig. 5a for irradiation of water with a beam of DLA electrons. This rapid oxygen depletion, induced by ultra-high dose rates, contrasts with the behavior at low dose rates, characteristic of linear accelerators (LINACs) used as X-ray sources, where a gradual decline is typically observed^31^. Although the expected drop in the measured molecular oxygen concentration should occur within ~ 1 ps, the measurements show that it was observed within the first second and continues to decrease for several more seconds, accompanied by noticeable signal fluctuations (Fig. 5a). This discrepancy can be explained by two factors. First, the temporal resolution of the oxygen meter is 1 s^31^. Second, the sensor measures the oxygen level in the near-surface layer of the sample, and the diffusion of oxygen molecules from the water layer into the polymer layer of the sensor requires several seconds to reach concentration equilibrium.

Figure 5b presents the dependence of the molecular oxygen concentration drop on the dose deposited by the DLA electrons. This dependence can be approximated by a linear fit with a slope of 0.35 ± 0.05 µM/Gy for water, based on four laser shots in the performed experiment (see blue dashed line in Fig. 5b), and 0.37 ± 0.05 µM/Gy for the culture medium, based on three laser shots (see green dashed line in Fig. 5b). For these approximations, the point with coordinates (0, 0) was fixed, which is based on physical considerations.

The slope of the linear fit for water, derived from experimental data, is slightly higher than the value predicted by simulations. This deviation is attributed to the dose reconstruction method, which exclusively accounts for the electron contribution. In practice, a correction for the X-ray dose is required. Based on background radiation measurements with RCF#1 (~ 10 Gy in shot #9) and RCF#2 (~ 1 Gy in shot #9), the X-ray dose in the water for shot #9 was estimated to be about 4 Gy, while the dose caused by electrons and X-rays in this shot was 40 Gy. Taking into account the contribution of electrons and X-rays, the recalculated slope of the linear fit is 0.32 ± 0.05 µM/Gy.

Experimental data presented in Fig. 5b are shown with error bars: the 10–15% error along the X-axis is due to the non-uniformity of the dose distribution within the volume of the tested sample (see e.g. the dose distribution at the RCF#2 relative to the tank position in Fig. 3). Better adjustment of the tank position relative to the electron beam would reduce this error. Along the Y-axis, the 7–10% error is determined by the noise level of the oxygen meter. Since it displays the averaged value over the measurement area, the error caused by the non-uniformity of the oxygen concentration is not considered. For more details related to the inhomogeneous dose distribution within the measurement area see “Supplementary Information”.

The obtained results were compared with Monte Carlo track structure simulations, which model the physical-chemical stage of electron and ion tracks in water. Such simulations are useful for calculation of the radiochemical yields in the early stages of radiation-induced chemical evolution. In this work, the experimental results are compared with predictions of the TRAX-CHEM code^13^. This code calculates the yields of molecular species and radicals in oxygenated aqueous solutions and has been used to estimate molecular oxygen removal as a function of the dose delivered by 1 MeV electrons in sealed oxygenated solutions. The calculated yield of molecular oxygen consumption in water is ~ 0.33 µM/Gy for initial oxygen concentrations ranging from 100 to 275 µM^14^. In^14^, it was shown that the linear fit is applicable for dependence of the molecular oxygen concentration drop on the dose deposited in water (see red line in Fig. 5b).

Based on the Monte Carlo simulations reported in^14^, it was concluded that within the dose range of 0 to 50 Gy, oxygen consumption is proportional to the absorbed dose. Owing to the linear nature of this relationship, it can be shown mathematically that this proportionality can be extended to the case of a non-uniform dose distribution by considering the average decrease in molecular oxygen concentration within the measured sample volume as a function of the average deposited radiation dose in that volume. Therefore, the transition to average dose values in the experiment occurs without any loss of physical meaning.

The experimentally obtained yield of molecular oxygen consumption in water, accounting for the aforementioned X-ray correction (0.32 ± 0.05 µM/Gy), is in good agreement with the predictions from the TRAX-CHEM code^13,14^(~ 0.33 µM/Gy). It is particularly noteworthy that, in the experiment, the dose delivered to the sample by high-energy electrons occurred within approximately 1 ps, which is consistent with the assumption used in Monte Carlo simulations. Under these conditions, the entire track evolution is completed within the first microsecond. In contrast, the irradiation times typically required in medical or FLASH accelerator facilities to achieve the high doses necessary for measurable oxygen depletion are of the same order of magnitude, or even longer, than the timescale of the simulated chemical stage.

### Setup optimization for future UHDR experiments

The pilot experiment conducted using the presented setup was associated with several challenges, such as accounting for the dose contribution from protons and X-rays, and the necessity of using a simulation-based dose reconstruction method. These issues were successfully addressed during the analysis and processing of the experimental results. Nevertheless, for prospective future experiments, it would be appropriate to introduce a series of simplifications and optimizations for the dose measurement. Specifically, this includes the removal of magnets and the use of a thicker plastic layer in front of the water tank to stop protons with energies ≤ 15 MeV and low-energy electrons (< 7 MeV), as well as to significantly reduce the X-ray contribution. Furthermore, the implementation of a symmetrical dose distribution approach in the two sections of the new setup (see below) – one containing the sample and the other containing the measuring RCFs – is proposed. This measure eliminates the need for dose reconstruction, allowing indirect measurements to provide more accurate results. Additionally, varying the laser target (combinations of foam with foil and PEEK) expands the dose range available for investigation. Further details are provided below.

**Fig. 6:**
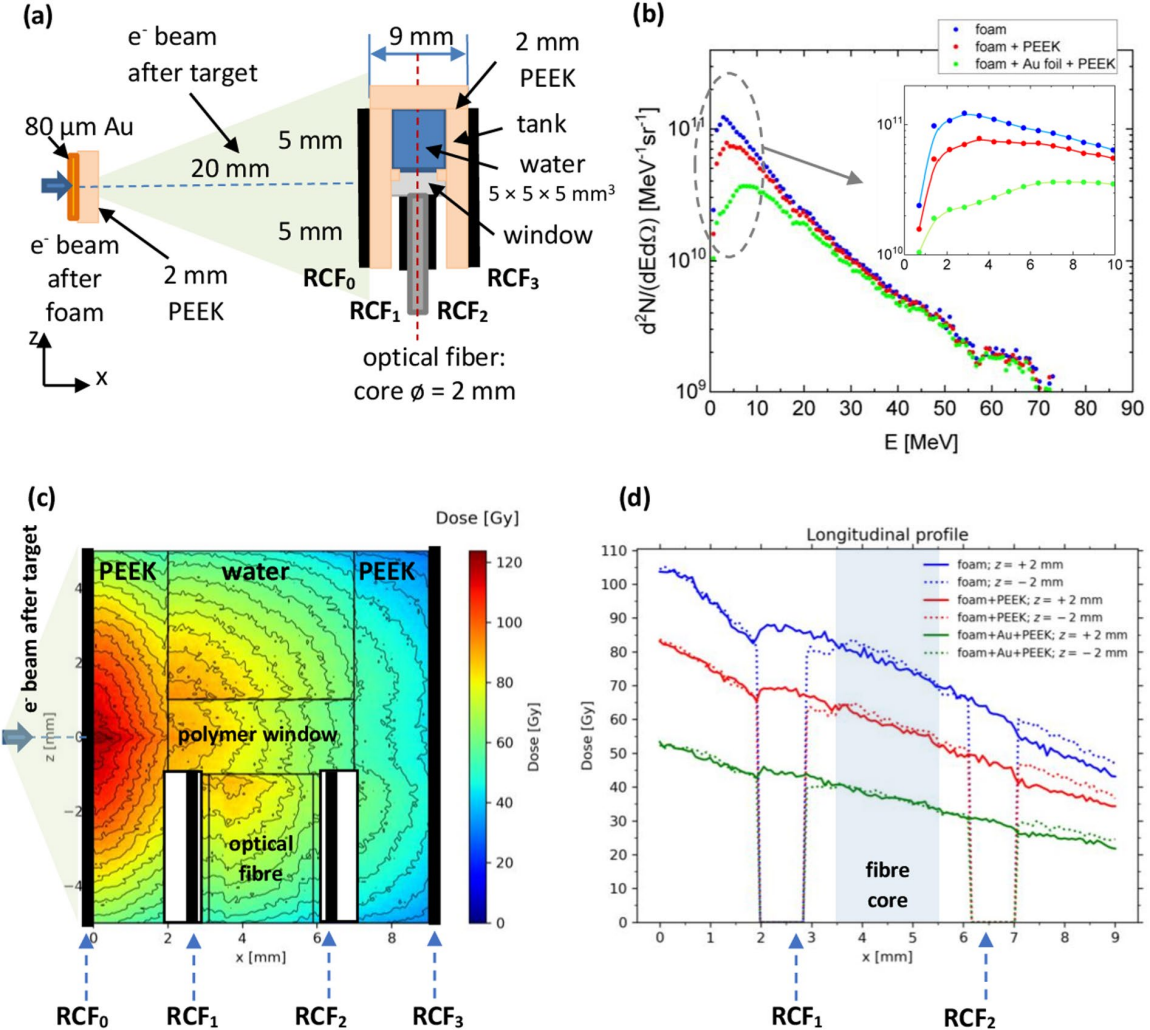
(**a**) Concept of the target setup. (**b**) Electron spectra after: foam (measured spectrum); foam + 2 mm PEEK; foam + 80 μm Au + 2 mm PEEK (simulated spectra), with a zoomed view of the low-energy region. (**c**) Dose distribution simulated for the conditions close to experiment using the new design of the tank in case of laser target “foam”. (**d**) Simulated distribution dose along x-axis (the laser/electron direction) for three electron-source setups at levels z = + 2 mm (water region near to the polymer window) and z = – 2 mm (symmetrical position along z-axis relative to the laser beam, in the measurement area).

The alternative design of the setup with the water tank is shown in Fig. 6a. In this setup, one half of the tank contains the liquid samples, while the second, open part houses RCFs positioned on the left and right sides of the optical fiber. Both parts share common 2 mm thick PEEK walls and are separated by a sealed optical window.

Simulations of dose distributions induced by electrons in water for the optimized setup were performed using the GEANT4 Monte Carlo code. The input parameters included electron energy and angular distributions measured during the interaction of a 10¹⁹ W/cm² PHELIX laser pulse with pre-ionized, 2 mg/cm³, 800 μm thick CHO foam. Three electron-source setups were considered: foam (case 1), foam + 2 mm PEEK (case 2), foam + 80 μm Au foil + 2 mm PEEK (case 3). Figure 6b shows the corresponding electron spectra obtained after these targets.

Figure 6c demonstrates simulated 2D dose distribution in the *XOZ* plane for the case 1 with foam as primary laser target. In the proposed scheme, the dose distribution is symmetric relative the laser/e-beam axis *OX* during the first 2 mm. As the lower part of the scheme contains vacuum spaces for placing the RCFs around the optical fiber, the dose measured at *z* = – 2 mm by RCF#1 will be slightly lower than that in water (*z* = + 2 mm) (Fig. 6d). This difference can be easily corrected during data evaluation.

As a result, we obtain the dose signals for cases 1, 2, 3 in the region that is relevant for measurements of the oxygen drop presented in Table 3. At the same time, it was taken into account that a change in the position along the *OY*-axis from − 2 mm to + 2 mm leads to a slight variation in the dose distribution for fixed *x* and *z* coordinates within the tank.

**Table 3:** Comparison of the simulated doses for different target cases in the relevant water measurement region and the corresponding RCF measurement region.

Target case	$\:{D}_{1}\:$[Gy], average dose in RCF_1_ for z = – 2 mm, y ∈ [−2 mm, + 2 mm]	$\:{D}_{1}\:$[Gy], dose in RCF_2_ for z = – 2 mm, y ∈ [−2 mm, + 2 mm]	$\:{D}_{av}=\frac{{D}_{1}+{D}_{2}}{2}$ [Gy], average dose in RCFs	$\:{D}_{w}\:$[Gy], average dose in water for z = – 2 mm, x ∈ [3.5 mm, 5.5 mm], y ∈ [−2 mm, + 2 mm]
1	85	70	78	78 ± 5 (± 6%)
2	67	52	60	60 ± 5 (± 8%)
3	45	32	39	39 ± 4 (± 10%)

The proposed new design eliminates the proton contribution and enables measurement of the deposited dose inside the tank under conditions very similar to those in water. The dose can be easily varied from 80 Gy down to 40 Gy per shot (ps) by changing the electron source configuration (1–3).

## Conclusion

In this study, we have demonstrated that laser-driven relativistic electron bursts offer distinct physical and radiobiological advantages over conventional sources. In contrast to protons, these beams of relativistic electrons maintain ultra-short pulse durations and therefore ultra-high dose rate by transport over significant distances. On the other hand, compared to X-rays, they allow for a higher energy deposition in matter.

By utilizing direct laser acceleration (DLA)of electrons in low density foams converted into plasma, we achieved high-current MeV electron beams capable of delivering doses up to 1–2 Gy per joule of focused laser energy. Specifically, using the PHELIX laser, we demonstrated single-shot doses of 20–50 Gy deposited into water. Crucially, the electron beam charge scales more than linearly with laser energy when accounting for increased intensity, which suggests a clear path toward even higher dose rates.

The high dose value and picosecond pulse duration result in an unprecedented dose rate of 7 × 10^13^ Gy/s. This allows for the temporal separation of instantaneous oxygen ionization from subsequent chemical reactions – a key requirement for investigating the underlying mechanisms of FLASH radiotherapy. Our observation of an abrupt, dose-correlated drop of molecular oxygen concentration in both water and culture media during each shot confirms the source’s capability to trigger FLASH-related processes.

Consequently, this laser-driven approach provides a unique platform for benchmarking Monte Carlo trace structure codes and the further development of FLASH radiotherapy.

## Supplementary Information

Below is the link to the electronic supplementary material.

Supplementary Material 1

## Data Availability

The data supporting the plots of this article and other results of this study are available from the corresponding authors on reasonable request.
