# Supplementary material for: Ultra-intense pulsed source of ionizing radiation based on direct laser acceleration of electrons for studying the FLASH effect
Source: Sci Rep. 2026 Feb 17;16:7164. doi: 10.1038/s41598-026-40281-4 (PMC12921297; doi:10.1038/s41598-026-40281-4)
Supplement: Supplementary file 1 — Supplementary Material 1 [file 41598_2026_40281_MOESM1_ESM.pdf]

Supplementary Information

Reconstruction of the dose inside the water tank based on DLA electrons

Simultaneous direct measurements of the molecular oxygen concentration and the dose inside the tank were not feasible, nor was it possible to measure the energy and angular distribution of the electrons generated in the laser-foam interaction for the configuration with both the magnet and the tank. Furthermore, the signal recorded on the first RCF positioned in front of the tank was dominated by protons (see main text, Fig. 4), which precluded any meaningful comparison between the RCF signals obtained at the front and rear of the tank.

To address these limitations, a reconstruction method was developed to determine the dose at six predefined positions on RCF #2 and RCF #3 (see Fig. 3 in the main text) by optimizing the adapted energy and angular electron distributions. Once the optimal distribution was established, the dose deposited by electrons in the target measurement area could be reliably determined.

1. Justification for the dose assessment with a dominant contribution from DLA electrons

For the GEANT4 simulations of the interaction between the electron beam and the water tank, electron spectra obtained from the interaction of a PHELIX laser pulse with pre-ionized CHO-foam (density 2 mg/cm³, thickness 800 μm) were used:

$$f = \frac{d^2N}{dE\cdot d\Omega}|_{\alpha,\beta=0^\circ} = 1.06\cdot 10^{12}\cdot\left(0.75\cdot exp\left(-\frac{E}{0.71}\right) + 0.25\cdot exp\left(-\frac{E}{9.85}\right)\right)[MeV^{-1}sr^{-1}]. \quad \text{(S1)}$$

The photon spectrum generated after passing through 5 mm of medium (PEEK, Mylar, or water) was simulated at a distance of 5.5 cm from the foam target. Results show that the X-ray dose in the RCF (EBT-XD) does not exceed ~1 mGy. Even the dose from all secondary particles (including secondary electrons and positrons) remains very low, at ~15 mGy.

The estimated dose from betatron radiation produced by DLA electrons in the relativistic plasma channel is significantly higher – about 20 Gy at a distance of 2 cm from the laser-foam interaction point. However, since these X-rays are prominent mainly in the 5–10 keV range [29], they are strongly attenuated after passing through 5 mm of medium (PEEK, Mylar, or water), yielding a dose of only ~0.04 Gy.

Additional to mentioned X-rays, there is also bremsstrahlung radiation from electrons passing through the carbon collimator. It was estimated on background radiation measurements with RCF#1 and RCF#2 that the X-ray dose contribution in water is ~ 10% (in shot #9 – 4 Gy).

These findings confirm that high-energy electrons have a dominant contribution to the dose in the medium inside or behind the tank. However, the correction for the dose from bremsstrahlung radiation should also be taken into account.

2. Description of the dose reconstruction method

To estimate the dose distribution with a dominant contribution from DLA electrons, the measured electron spectra [12, 25] in the following form were used:

$$f = \frac{d^2N}{dE\cdot d\Omega}|_{\alpha} = N_0\left(\lambda\cdot exp\left(-\frac{E}{T_1}\right) + (1-\lambda)\cdot exp\left(-\frac{E}{T_2}\right)\right)\cdot exp\left(-\left(\frac{\alpha-\alpha_0}{\Delta\alpha}\right)^2\right), \quad \text{(S2)}$$

where N_0 is normalization factor in $MeV^{-1}sr^{-1}$, λ and $1-\lambda$ are the fractions of particles with temperatures T_1 and T_2, α_0 is the angle between the laser axis and the electron beam, $\Delta\alpha$ is the divergence angle parameter (half-angle of divergence: $\Delta\alpha_{1/2FWHM} = \Delta\alpha\cdot\sqrt{ln\ (2)}$).

2.1. Method implementation in Python

To reconstruct the dose distribution in the tank medium, the geometry of the magnetic assembly, the tank, and their relative positions to the foam target were first defined. Using the known magnetic field map of the setup (Fig. 2a, main text) and accounting for both deflection of par-

ticles in the magnetic field and their energy loss when passing through the medium, the trajectories of electrons with different initial energies and emission angles relative to the target normal were calculated.

In a stepwise computation, the kinetic energy E_i and incidence angle α_i of electrons entering the medium were determined for specific positions in the measurement area, based on the initial parameters E_{0i} and α_{0i} at the laser target.

By evaluation for different positions in the measurement area with small spatial increments, the resulting relations:

$$E_i = E_i(E_{0i}, \alpha_{0i}), \qquad \alpha_i = \alpha_i(E_{0i}, \alpha_{0i}),$$

were mapped as functions of the spatial coordinates

$$\mathbf{r}_j = \begin{bmatrix} x_j \\ y_j \\ z_j \end{bmatrix} = \mathbf{r}_j(\mathbf{r}_{0i}, \mathbf{v}_{0i}, E_{oi}).$$

These mappings form matrices that enable dose calculations for a given initial electron energy distribution at the laser target.

2.2. Optimization procedure

As an initial guess for the electron spectrum at the foam target, data from a laser shot with the same target parameters were used. This spectrum was propagated to the six measurement regions (1-6 in Fig. 3, main text) using the calculated transfer matrices.

The difference between the simulated and measured doses in these positions was minimized by adjusting the parameters of the distribution function (S2) through an optimization routine.

2.3. Application to shot #9 P213

As a representative case, we examined shot #9 P213, where the target consisted of an 800 µm CHO-foam with a density of 2 mg/cm³. The same target parameters were used in shot #25 P207 from a separate experiment ("Betatron radiation measurement"), conducted with an identical experimental setup. In that experiment, electron spectra were recorded at multiple angular positions, providing the initial input for the dose reconstruction in shot #9.

The optimization process aimed to minimize the difference between simulated and experimentally measured dose values in regions 1–6 (marked in Fig. 3, main text). This was achieved by running 100 iterations of parameter variations in the electron distribution function (S2) using a Python-based routine.

The optimized spectrum for shot #9 P213 is shown in Fig. 1S and is described by the following expression:

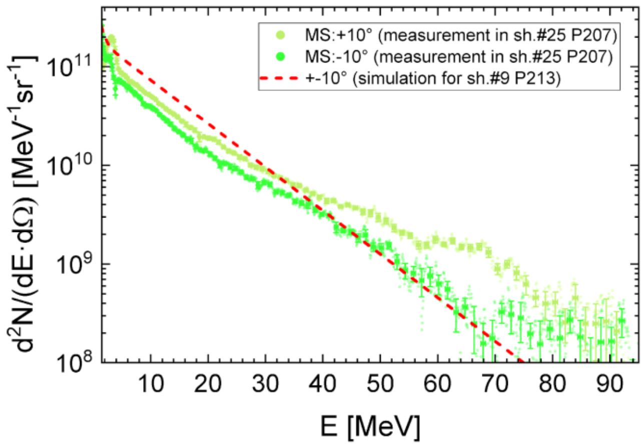

Fig. 1S. Comparison between the measured electron spectrum from shot #25 P207 at +10° relative to the laser axis and the simulated spectrum for shot #9 P213. Both cases correspond to the same target configuration: 800 ± 50 µm CHO-foam with a density of 2 mg/cm³.

$$f = \frac{d^2N}{dE \cdot d\Omega}|_\alpha = 1.06 \cdot 10^{12} \cdot \left(0.75 \cdot exp\left(-\frac{E}{0.71}\right) + 0.25 \cdot exp\left(-\frac{E}{9.85}\right)\right) \cdot exp\left(-\left(\frac{\alpha - 6.71}{6.3}\right)^2\right) \quad \text{(S3)}$$

with units $[f] = MeV^{-1}sr^{-1}$, $[E] = MeV$, $[\alpha] = °$.

3. Verification of results of the reconstruction method

Several shots were conducted with RCFs placed inside the tank to validate simulated dose values against direct measurements. In shot #9 (P213), mini-RCFs were positioned inside the tank and irradiated with a DLA beam. The resulting data (Fig. 2S) enabled verification of the optimized electron distribution for this shot.

Using the optimized electron distribution function, the calculated dose in the measurement region inside the tank was 40 ± 7 Gy (±18 %). The corresponding experimental measurement from the middle RCF yielded 44 ± 4 Gy (±11 %) (Fig. 2S). With a correction for the fact that the dose calculated based on DLA electrons does not account for the ~10% contribution from bremsstrahlung radiation (~4 Gy in shot #9 (P213)), there is good agreement between the simulation and the measurements, confirming the validity of the reconstruction method.

The same approach was successfully tested in other shots. It was subsequently applied to cases where the tank was filled with a water-like liquid, making direct dose measurements inside the tank impossible. In these cases, the measurement area inside the tank was instead monitored via molecular oxygen concentration measurements using an optical fiber.

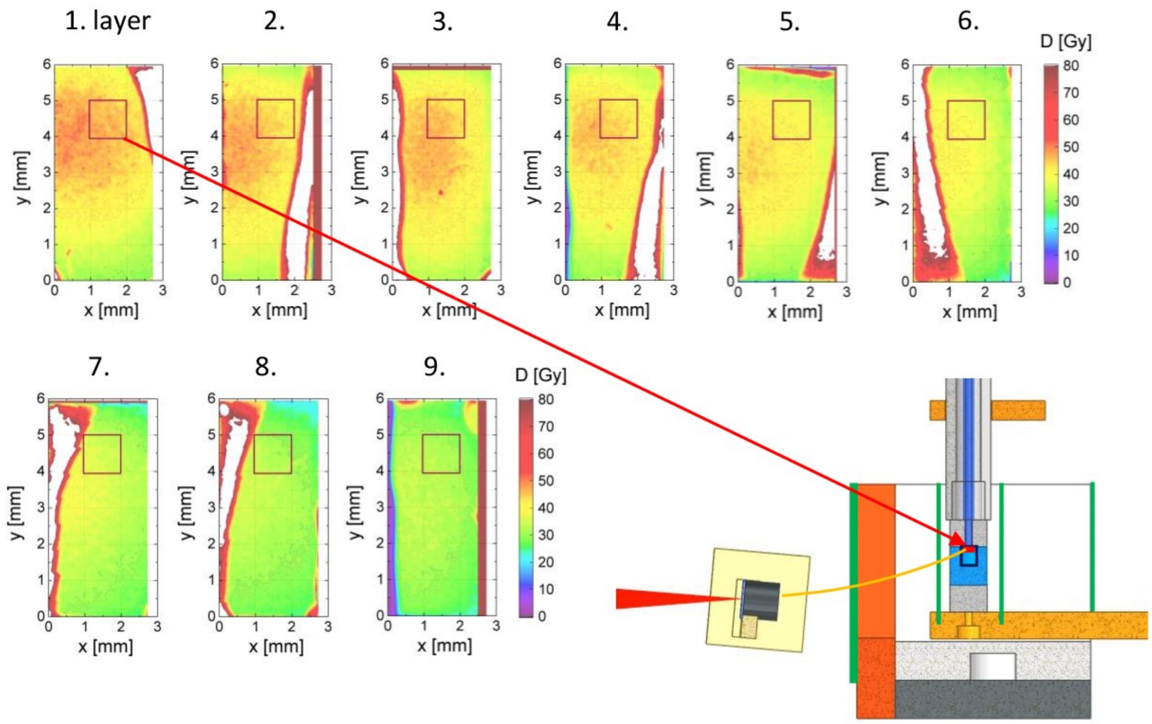

Fig. 2S. Three-dimensional dose distribution in the water tank, measured using mini-RCFs placed inside the tank to validate the dose reconstruction method for shot #9. The red box indicates the 1 × 1 mm² measurement area. All RCFs exhibit edge signal distortions due to their delamination caused by cutting with scissors.
